# Myoclonus as a neurologic manifestation of hypoglycemia in dogs

**DOI:** 10.1093/jvimsj/aalag252

**Published:** 2026-10-13

**Authors:** Dakir Polidoro, Theofanis Liatis, Patricia Álvarez, Sofie F M Bhatti

**Affiliations:** AniCura Veterinary Practice Plantijn, 2600 Antwerp, Belgium; AniCura Vetimacs Medical Imaging, 1060 Brussels, Belgium; Plakentia Veterinary Clinic – Alimos, 17455 Athens, Greece; School of Veterinary Medicine, European University Cyprus, 1516 Nicosia, Cyprus; Independent Vetcare Evidensia, Pride Veterinary Referrals, DE24 8HX Derby, United Kingdom; Small Animal Department, Faculty of Veterinary Medicine, Ghent University, 9820 Merelbeke-Melle, Belgium

**Keywords:** movement disorder, cortical–subcortical myoclonus, head jerks, insulinoma, neuroglycopenia, canine

## Abstract

**Background:**

Myoclonus consists of brief, shock-like jerks and is not reported in dogs as a neurological manifestation of hypoglycemia.

**Hypothesis/Objectives:**

To describe hypoglycemia-associated myoclonus in dogs and report diagnostic findings, treatment, and outcome.

**Animals:**

Ten dogs presenting myoclonus and concurrent hypoglycemia.

**Methods:**

Multicenter, retrospective study of dogs presenting hypoglycemia-related myoclonus.

**Results:**

All dogs presented with myoclonus. Neurological examination was abnormal in 5/10 (50%). Myoclonus was generalized in 8/10 (80%) dogs and focal (head) in 2/10 (20%), with progression to generalized tonic–clonic epileptic seizures in 5/10 (50%). Hypoglycemia (<3.3 mmol/L) was observed in all dogs (median, 2.07 mmol/L) during the myoclonic episodes. Insulinoma was a suspected (*n* = 1), presumptive (*n* = 5), or definitive (*n* = 3) diagnosis in 9/10 (90%) dogs. One dog (10%) developed iatrogenic hypoglycemia after IV administration of fast-acting human insulin and myoclonic episodes resolved after dose reduction. Among dogs with suspected, presumptive or confirmed insulinoma, 5/9 (56%) received medical treatment alone, 3/9 (33%) combined medical and surgical treatment, and 1/9 (11%) was not treated. Medical treatment included, prednisolone (*n* = 6), diazoxide (*n* = 3) and therapeutic diet (*n* = 6). Surgical treatment consisted of pancreatic mass resection. Medical or surgical treatment resulted in short-term improvement in myoclonus frequency in all dogs, however, four were later euthanized due to recurrence of hypoglycemia and myoclonus.

**Conclusions and clinical importance:**

Myoclonus might represent an underrecognized neurological manifestation of hypoglycemia in dogs. Measurement of blood glucose concentrations is a fundamental diagnostic approach for identifying hypoglycemia-related myoclonus. Treatment can lead to clinical improvement, but prognosis is dependent on the underlying cause.

## Abstract (Dutch)

**Achtergrond:**

Myoclonus wordt gekenmerkt door korte, schokachtige spiercontracties en werd bij honden niet eerder beschreven als neurologische manifestatie van hypoglycemie.

**Doelstelling:**

Beschrijven van hypoglycemie-geassocieerde myoclonus bij honden en diagnostische bevindingen, behandeling en klinische uitkomst rapporteren.

**Dieren:**

Tien honden met myoclonus en hypoglycemie.

**Methoden:**

Multicentrisch retrospectief onderzoek bij honden met hypoglycemie-geassocieerde myoclonus.

**Resultaten:**

Alle honden presenteerden myoclonus. Bij 5/10 (50%) honden was het neurologisch onderzoek afwijkend. Myoclonus was gegeneraliseerd bij 8/10 (80%) honden en focaal (hoofd) bij 2/10 (20%). Bij 5/10 (50%) honden trad progressie op naar gegeneraliseerde epilepsieaanvallen. Bij alle honden werd tijdens de myoclonische episoden hypoglycemie (<3,3 mmol/L; mediane 2.07 mmol/L) vastgesteld. Insulinoma was de vermoedelijke (*n* = 1), waarschijnlijke (*n* = 5) of definitieve (*n* = 3) diagnose bij 9/10 (90%) honden. Een hond (10%) ontwikkelde iatrogene hypoglycemie na intraveneuze toediening van humane insuline. Van de 9 honden met vermoedelijke of bevestigde diagnose van insulinoma werden 5/9 (56%) medicamenteus behandeld, 3/9 (33%) kregen een combinatie van medicamenteuze en chirurgische behandeling en 1/9 (11%) werd niet behandeld. Medicamenteuze behandeling bestond uit prednisolone (*n* = 6), diazoxide (*n* = 3) en therapeutisch dieet (*n* = 6). Chirurgische behandeling bestond uit pancreasmassa resectie. Medicamenteuze en chirurgische behandeling resulteerde bij alle honden verbetering op korte termijn van de myoclonus frequentie. Vier honden werden later geëuthanaseerd wegens recidief van hypoglycemie en myoclonus.

**Conclusies en klinische relevantie:**

Myoclonus kan een onderkende neurologische uiting van hypoglycemie bij honden zijn. Het bepalen van de bloedglucoseconcentratie vormt een essentiële diagnostische stap. Behandeling van hypoglycemie kan leiden tot klinische verbetering, maar prognose is afhankelijk van de onderliggende oorzaak.

## Introduction

Myoclonus is characterized by a sequence of repeated, short-lasting, shock-like jerks, resulting from sudden involuntary contraction (positive) or relaxation (negative) of one or more muscles, that will generate movement of the affected body part.^1^^,^^2^ In humans, a new classification for myoclonus has been recently introduced, which includes clinical features, anatomical distribution, neurophysiological markers, and etiology of myoclonus.^3^ This classification is structured around 2 axes: Axis I provides a detailed clinical characterization of patients with myoclonus, and Axis II addresses the etiology.^3^ Furthermore, Axis I is subdivided into Axis Ia and Ib. Axis Ia encompasses historical features, myoclonus characteristics, and diagnostic evaluation, whereas Axis Ib includes clinical neurophysiological features of myoclonus. Within Axis Ib, an anatomo-physiological classification of myoclonus is proposed, comprising the following subtypes: cortical, cortical–subcortical, subcortical, brainstem, spinal, propriospinal, and peripheral.^3^ In humans, myoclonus has a wide range of etiologies, including genetic, acquired, functional, idiopathic, and physiologic causes. Among these, acquired etiologies have historically been most frequently reported and include conditions such as metabolic, toxic- and drug-induced causes, and nervous system lesions.^3^ Metabolic and toxic- and drug-induced myoclonus are presumed to be of cortical–subcortical origin and are often reversible after removing the offending agent or correcting the underlying metabolic disturbance, including disorders such as hypoglycemia, hyperglycemia, renal and hepatic failure and hyponatremia.^3^^,^^4^ Nervous system lesions represent another important cause of myoclonus. Among these, hypoxic brain injury is one of the most well-described etiologies in humans and might occur in both acute and chronic forms.^3^^,^^5^

In veterinary medicine, myoclonus is simply classified according to its topographic distribution (focal or generalized) and its presumptive association with epilepsy (epileptic or nonepileptic myoclonus).^1^^,^^2^ In dogs, several etiologies of myoclonus have been reported, such as degenerative encephalopathy-associated myoclonus (eg, Lafora disease, neuronal ceroid lipofuscinosis),^1^ myoclonic epileptic seizures (eg, juvenile myoclonic epilepsy associated with *DIRAS1* gene mutation, spontaneous late-onset myoclonic epilepsy),^6^^,^^7^ drug-induced myoclonus (eg, opioids, propofol),^8–10^ encephalitis-associated myoclonus (eg, canine distemper virus infection),^11^^,^^12^ intoxication (eg, fluralaner, marijuana),^13^^,^^14^ cervical myoclonus (due to cervical myelopathies)^15^^,^^16^ and acute post-hypoxic myoclonus.^17^

Although numerous etiologies have been associated with myoclonus in dogs, hypoglycemia has not previously been reported as an underlying cause. The aim of this study was to describe, for the first time, myoclonus as a neurologic manifestation of hypoglycemia in dogs and to report the associated diagnostic findings, treatment, and outcome.

## Materials and methods

This is an observational retrospective, multicenter, case series study of dogs presenting myoclonus as a neurologic manifestation of hypoglycemia. Ethical approval was not necessary due to the retrospective nature of this study.

Cases were collected among colleagues that have anecdotally reported potential cases of myoclonus in dogs due to hypoglycemia. Inclusion criteria were (1) complete medical records, (2) dogs presenting with episodes of myoclonus with an acute onset, with or without any other concurrent signs of neurological disease, while on a hypoglycemic state, defined as a blood glucose concentration below 3.3 mmol/L, and (3) with a video footage available of the episodes. Exclusion criteria included (1) insufficient characterization of the clinical signs and absence of video footage of the episodes, (2) blood glucose measurements performed using a human-specific portable glucometer, and (3) blood samples that were either not immediately analyzed using an in-house veterinary point-of-care chemistry analyzer or, if submitted to an external laboratory, not collected in sodium fluoride tubes.

Clinical information for each case was obtained from the medical records and included signalment, medical history, clinical and neurological examination, myoclonus semiology (occurrence and characterization of episodes based on video footage and owners’ description), diagnostic test findings, treatment and outcome. For myoclonus semiology, evaluated characteristics included the distribution of affected body regions, posture and mentation during episodes, distractibility and potential progression to focal or generalized epileptic seizures. Myoclonus was considered epileptic when such progression was observed. Diagnostic tests included CBC and serum biochemistry profile (including glucose concentrations), abdominal ultrasound, computed tomography (CT), magnetic resonance imaging (MRI), and cerebrospinal fluid (CSF) analysis. For each dog, the blood glucose concentration reported was the lowest measured pre-treatment value obtained before initiation of medical or surgical treatment of the underlying cause of hypoglycemia. For the diagnostic criteria of insulinoma, dogs were assigned a presumptive diagnosis when findings were considered highly suggestive of insulinoma, including (1) imaging findings (abdominal ultrasound or CT) consistent with a pancreatic mass suggestive of insulinoma in the presence of documented hypoglycemia and serum insulin concentrations within or above reference intervals, or (2) documented hypoglycemia accompanied by increased serum insulin concentration, with no abnormalities detected on imaging examination. Dogs were assigned a definitive diagnosis of insulinoma when histopathological confirmation was available.^18^ Dogs that did not fulfill these criteria (hypoglycemia only, with no abnormal imaging findings) were assigned a diagnosis of suspected insulinoma. The outcome and follow-up were exclusively based on clinical records or communication logs. Improvement was defined as remission or a reduction in the frequency of the myoclonus and other signs of neurological disease.

## Results

### Animals

Ten dogs met the inclusion criteria and no dogs were excluded. Cases were collected from 4 different veterinary institutions and private hospitals across Belgium (*n* = 9) and United Kingdom (*n* = 1). Five (50%) dogs were male (3 neutered) and 5 (50%) were female (4 neutered). The median age at onset of myoclonus was 109 months (range, 24 to 152 months). Breeds included Border Collie (3/10; 30%) and one of each below: (1/10; 10%) Australian Shepherd, Belgian Malinois, Bernese Mountain Dog, Braque Français, French Bulldog, Jack Russel terrier and Shih Tzu. Median body weight was 18 kg (range, 7.2 to 40 kg). Information regarding diet when the episodes started was available for all dogs and most of them (9/10; 90%) were receiving commercial maintenance dog food, while 1/10 (10%) dog was receiving a hypoallergenic diet.

### Presenting complaints

All dogs were presented due to myoclonic episodes of acute onset. During the episodes at home, owners reported obtundation 8/10 (80%), generalized body tremors 6/10 (60%) and weakness 5/10 (50%).

### Clinical and neurological findings

No abnormalities were detected on clinical examination, except for tachypnea (3/10; 30%) and vaginal bleeding related to a small and irregular lesion at the vaginal vestibule (1/10; 10%). Neurological examination was normal in 5/10 (50%) dogs and abnormal in the other 5/10 (50%). Neurological abnormalities found were myoclonus (5/5; 100%), obtundation (4/5; 80%), ambulatory tetraparesis (4/5; 80%), reduced to absent menace response bilaterally (4/5; 80%) and reduced to absent postural reactions in all limbs (3/5; 60%). All dogs had neuroanatomical localization consistent with a forebrain lesion, with 5/10 (50%) showing multifocal localization involving the forebrain and brainstem. Neuroanatomical localization was not based solely on abnormalities identified during the neurological examination, but also on abnormalities observed in the video recordings. This approach also included dogs that were neurologically normal at the time of examination but exhibited signs of neurological disease in the recorded episodes. Neurological examination and video recordings analysis in all cases were performed by a board-certified neurologist or a neurology resident under the direct supervision of a board-certified neurologist.

### Characterization of the episodes

Semiology of the myoclonic episodes included the whole body (generalized) in 8/10 (80%) and only the head (focal) in 2/10 (20%) dogs (Video 1). Regarding posture, myoclonic episodes could be observed during rest (lateral or sternal decubitus) and while standing or walking in 7/10 (70%) dogs (Video 1). In 3/10 (30%) dogs, episodes were only observed while in lateral decubitus. Tendency to fall back, over or to the side, during the myoclonic episodes, was reported in 7/10 (70%) dogs (Video 1). Daily episodes were reported in 2/10 (20%) dogs, but information regarding episode duration was unavailable for both dogs. One dog was reported to have experienced a single myoclonic episode sequence lasting from 5 to 10 min. For the remaining dogs, precise information on episode frequency and duration was lacking, although both were reported to be highly variable within affected individuals. Myoclonic episodes were not distractible by tactile or auditory stimuli in any dog. However, some degree of environmental awareness was preserved in all of them, with 4/10 (40%) appearing fully conscious during the episodes. No signs of autonomic dysfunction were observed during myoclonic episodes. In 5/10 (50%) dogs, myoclonus could progress to generalized tonic–clonic epileptic seizures. Autonomic manifestations, including urination and hypersalivation, were observed exclusively during generalized tonic–clonic epileptic seizures and were also accompanied by loss of consciousness.

### Diagnostic evaluation

Complete blood count and serum biochemistry profile were performed in all dogs. Blood glucose concentrations after feed withdrawal were available in all cases and were below 3.3 mmol/L in every dog. Median blood glucose concentration after feed withdrawal was 2.07 mmol/L (range; 1.1-3.2 mmol/L). In 9/10 (90%) dogs, the blood sample was obtained while the myoclonic episodes were present, either by the referring veterinarian or by the neurologist. Insulinoma was initially suspected in 9/10 (90%) dogs. Of these, 8/9 (88.9%) underwent CT scan of the thorax and abdomen, whereas 1/9 (11.1%) was evaluated using abdominal ultrasonography alone. Among dogs that underwent CT scan of the thorax and abdomen, no abnormalities were detected in 3/8 (37.5%), while 4/8 (50%) had nodular thickening of a pancreatic lobe and 1/8 (12.5%) a bigger pancreatic mass lesion. All 5 cases were consistent with insulinoma. In one of the dogs diagnosed with a pancreatic nodule, a pulmonary nodule within the right cranial lung lobe was also identified and considered compatible with metastasis. This was the same dog that presented with vaginal bleeding on clinical examination. Biopsy of the lesion within the vaginal vestibule revealed a neoplasm of mucosal epithelial origin, most consistent with carcinoma. In the dog evaluated only by abdominal ultrasonography, a pancreatic nodule compatible with insulinoma was also identified.

The only dog that did not undergo imaging examination was presented to the emergency service due to a window entrapment trauma in the left thoracic limb. This dog was the sole case with an abnormal CBC, characterized by mild leukocytosis, and exhibited multiple biochemical abnormalities, including markedly high serum creatinine (764 μmol/L; RI, 44-159 μmol/L) and urea (40.8 mmol/L; RI, 2.5-9.6 mmol/L), markedly high creatine kinase (CK; unmeasurable despite repeated dilution attempts), high aspartate aminotransferase (AST; > 1,000 U/L; RI, 10-125 U/L) and alkaline phosphatase (ALP; 327 U/L; RI, 23-212 U/L), and hyperkalemia (7.1 mmol/L; RI, 3.7-4.7 mmol/L). To correct the hyperkalemia, the dog received multiple glucose boluses followed by a constant rate infusion of glucose 5% (4 mL/kg/h), in combination with IV administration of fast-acting human insulin (Actrapid, Novo Nordisk; 0.1-0.4 IU/kg). The dog acutely developed an abnormal mental status (obtundation) and episodes of generalized myoclonus. At that time, blood glucose concentration was measured and found to be low (2.7 mmol/L). Based on this, iatrogenic hypoglycemia secondary to fast-acting insulin administration was suspected.

Insulin concentrations after feed withdrawal were measured in 5/9 (56%) dogs with suspected insulinoma at the time of hypoglycemia and were increased in 4/5 (80%), with a median concentration of 792 pmol/L (range, 67-900 pmol/L; RI, 12-126 pmol/L). Fructosamine concentrations after feed withdrawal were assessed in 3/9 (33%) dogs and were unremarkable.

In the 6 dogs in which insulinoma was initially suspected on the basis of imaging findings, either via abdominal CT scan (*n* = 5) or ultrasonography (*n* = 1), histologic confirmation was obtained via biopsy in 3/6 (50%) cases. According to the predefined diagnostic criteria for insulinoma, one dog was classified as suspected insulinoma, 5 dogs as presumptive insulinoma, and 3 dogs as having a definitive diagnosis of insulinoma confirmed by histopathology. Magnetic resonance imaging of the brain was performed in 2/10 (20%) dogs and cerebellomedullary cistern CSF analysis was performed in 1/10 (10%) dog, all with no abnormalities. Electroencephalography (EEG) was not performed in any of the dogs.

### Clinical course, treatment, and outcome

No treatment was pursued in 1/10 (10%) dog, while 9/10 (90%) dogs received treatment based on the primary cause of the hypoglycemia. Follow-up information was available for all dogs, with a median time from presentation to follow-up of 3.5 months (range, 2 to 7 months).

In the dog with traumatic injury of the left thoracic limb, in which iatrogenic hypoglycemia was suspected, myoclonic episodes resolved completely after adjustment of the 5% glucose constant rate infusion and reduction of the fast-acting human insulin dose. Other laboratory abnormalities, including high creatinine, urea, CK, ALT, ALP, and potassium concentrations, normalized within several days of clinical management of the traumatic injury. The 5 dogs with a presumptive diagnosis of insulinoma received medical treatment alone, whereas the 3 dogs with a definitive diagnosis of insulinoma received a combination of medical and surgical treatment. The remaining dog, which was the only dog classified as having suspected insulinoma according to the predefined diagnostic criteria, did not receive any treatment and was euthanized at the owner’s request after being presented on emergency because of acute generalized tonic–clonic epileptic cluster seizures. Medical treatment included prednisolone (0.5-1 mg/kg PO q24h; *n* = 6), diazoxide (3.3 mg/kg PO q12h; *n* = 3) and therapeutic diet formulated for glycemic control (eg, Hill’s Prescription Diet w/d, Hill’s Pet Nutrition; *n* = 6). Also, all 8 dogs with a presumptive or definitive diagnosis of insulinoma that were treated, were fed multiple small meals daily to support glycemic stability. Surgical treatment consisted of resection of the pancreatic mass (*n* = 3).

Of the 5 dogs with presumptive diagnosis of insulinoma that received medical treatment alone, 3 were euthanized due to clinical deterioration associated with recurrent hypoglycemia and recurrence of signs of neurological disease, mainly myoclonic episodes. Two of these dogs, including the one diagnosed with carcinoma of the vaginal vestibule and pulmonary metastasis, remained episode-free for 3 months before being submitted to euthanasia. In the remaining dog that was also euthanized, myoclonic episodes never completely resolved, but frequency dramatically decreased for a period of 5 months. Of the remaining 2 dogs that received medical treatment only, one was episode free, whereas the other experienced a single episode of head myoclonus during follow-up periods of 3 and 5 months, respectively. Among dogs that underwent surgical resection of the pancreatic mass in addition to medical treatment, one was euthanized 6 months after surgery because of clinical deterioration associated with recurrent hypoglycemia, including recurrence of myoclonic episodes and generalized tonic–clonic epileptic seizures. This dog remained episode free for 3 months. Of the 2 remaining dogs that underwent combined medical and surgical treatment and were alive at the last follow-up record, one remained episode free at 7 months of follow-up, whereas the other remained episode free for four months before being lost to follow-up. One of these 2 dogs, developed transient hyperglycemia after surgery and was placed on a treatment with insulin.

Control glucose concentrations post-treatment, either medically or surgically, were available for 7/9 (77.8%) dogs, and were all within reference interval in the period that these dogs remained myoclonic episode free. Before the diagnosis of hypoglycemia, 4/10 (40%) dogs were treated with antiseizure medications (ASMs), including levetiracetam (20 mg/kg PO q8h) or phenobarbital (4 mg/kg PO q12h). Notably, none of these dogs responded to therapy, and all continued to experience generalized tonic–clonic epileptic seizures or myoclonus during the hypoglycemic state. As none of these dogs initially responded to antiseizure therapy and all showed improvement after correction of hypoglycemia, ASM were subsequently discontinued in all individuals. Information regarding the duration of ASM administration before discontinuation was unavailable for all four dogs.

## Discussion

This retrospective case series describes myoclonus as a neurologic manifestation of hypoglycemia in 10 dogs, with clinical signs improving after medical or surgical treatment of the underlying disease. In humans, hypoglycemia is commonly associated with signs of neurological disease such as behavioral changes, confusion, fatigue, tremors, cortical blindness, epileptic seizures, and coma.^19^ Similar manifestations are described in dogs, particularly those diagnosed with insulinoma, such as altered mental status, abnormal behavior, epileptic seizures, proprioceptive ataxia, weakness (paresis), collapse, visual deficits, tremors and twitches, including muscle fasciculations.^18^^,^^20–28^ Although tremors are frequently reported as a clinical sign in dogs with hypoglycemia, these involuntary muscle contractions might be more appropriately classified as twitches rather than tremors.^29^ Twitches arise as a consequence of peripheral nerve hyperexcitability, a motor neuron dysfunction that can manifest clinically as cramps, fasciculations, myokymia, neuromyotonia, tetany, and tetanus.^30^^,^^31^ Unlike true tremors, which are characterized by a regular, rhythmic, and sinusoidal oscillation of uniform frequency, twitches exhibit an irregular frequency, variable amplitude, and erratic pattern.^2^^,^^30^^,^^32^ Nevertheless, twitches resulting from peripheral nerve hyperexcitability can closely mimic tremors, making the distinction between the 2 clinically challenging. Consequently, the term tremor is sometimes used in the veterinary literature as an umbrella term encompassing both true tremors and twitches.^33^ On the other hand, although some overlap might exist between twitches and myoclonus, twitches resulting from peripheral nerve hyperexcitability can be distinguished from myoclonus by the absence of movement in the affected body segment.^29^^,^^30^^,^^32^ This is a report of myoclonus as a neurological manifestation of hypoglycemia in dogs and it is possible that myoclonus has been overlooked or underreported in previous studies. Indeed, differentiating myoclonus from other paroxysmal disorders can be challenging, particularly when not evaluated by a neurologist. Consequently, it is possible that myoclonus might have been misclassified as other involuntary movement in previous studies, such as tremors or twitches. Other uncommon signs potentially associated with hypoglycemia in dogs diagnosed with insulinoma, including suspected paroxysmal dyskinesia and head tilt, are reported.^18^^,^^28^ However, it remained unclear whether these atypical manifestations represented distinct conditions occurring concurrently with hypoglycemia, suggesting that these observations might have been incidental or unrelated findings rather than genuine clinical features of insulinoma-associated hypoglycemia. Neither suspected paroxysmal dyskinesia nor head tilt was observed in the dogs included in the present study. All clinical signs observed in the dogs in this study, including myoclonus, had an acute onset.

In humans, acute to subacute myoclonus is most commonly associated with metabolic disorders such as hypoglycemia^34^ and is presumed to be of cortical–subcortical origin.^4^ The pathophysiology of cortical–subcortical myoclonus is thought to involve widespread, abnormal bidirectional neuronal activity between cortical and subcortical circuits, resulting in diffuse excitation.^3^ Because excitation over the sensorimotor cortex is simultaneously widespread, the resulting myoclonus is typically generalized.^3^ In the present study, multiple signs of neurological disease were observed in all cases, either during neurological examination or on video recordings, however, acute myoclonus was the most consistent finding, occurring in all dogs and classified as generalized in 80%. Myoclonus was considered acute because of its abrupt onset, although it was recurrent in most dogs (90%). Unfortunately, due to the retrospective nature of the study, the precise frequency and duration of individual myoclonic episodes could not be determined in any of the dogs. In all dogs, the myoclonus was also considered reactive in nature, occurring secondary to the metabolic disturbance caused by hypoglycemia. Moreover, myoclonus occurred in various postures, including standing and during gait, which in some dogs resulted in transient falls. This is likely related to sudden alterations in consciousness caused by diffuse cortical excitability, a hallmark of cortical–subcortical myoclonus.^34^ In dogs experiencing multiple episodes, although precise information regarding the extent of this variability was not available, both frequency and duration were reported to be highly variable, likely reflecting differences in the time required to correct the hypoglycemic state as well as due to fluctuations in blood glucose concentrations, which can vary considerably throughout the day in dogs with insulinoma for example.^26^ These variations are most likely influenced by counter-regulatory mechanisms, such as glucagon secretion stimulation, and the effects of feeding through the day.^23^^,^^35^

Myoclonus as a clinical manifestation of hypoglycemia is reported in a few cases in humans,^36–41^ but has not been described in dogs. In a study in dogs diagnosed with spontaneous late-onset myoclonic epilepsy, one dog concurrently diagnosed with diabetes mellitus exhibited myoclonic episodes that appeared to worsen during times of hypoglycemia and did not completely resolve during normoglycemia.^7^ Nevertheless, a definitive association between hypoglycemia and the increased frequency of myoclonic episodes could not be established in this specific case.

In healthy dogs, normal blood glucose concentration is maintained within a narrow range (3.3-6.2 mmol/L), with hypoglycemia defined as a concentration below 3.3 mmol/L.^23^^,^^42^^,^^43^ All dogs in this study were diagnosed with hypoglycemia, with a median blood glucose concentration after feed withdrawal of 2.07 mmol/L. Artifactual hypoglycemia caused by sample handling or measurement artifacts is commonly described in dogs.^23^ To minimize the risk of including spurious hypoglycemic measurements in this study, only dogs with blood glucose concentrations obtained using veterinary-specific devices or appropriate sample handling procedures were included. Specifically, blood glucose measurements were all performed using a veterinary-specific portable glucometer or immediately analyzed using an in-house veterinary point-of-care chemistry analyzer. Alternatively, if samples were submitted to an external laboratory, they were collected in sodium fluoride tubes. Although clinical signs typically do not manifest until blood glucose concentrations fall below approximately 2.2 to 2.8 mmol/L,^23^ at least one dog in this group was still presenting spontaneous myoclonic episodes with a glucose level of 3.2 mmol/L. These signs of neurological disease are attributable to cerebral hypoglycemia, also termed neuroglycopenia.^43^

In our study, insulinoma was considered the underlying cause of hypoglycemia in most dogs (90%), including cases classified as suspected, presumptive, or definitive according to the predefined diagnostic criteria. In dogs with suspected or presumptive insulinoma, the diagnosis was based on signalment and clinical history together with the Whipple’s triad, which is defined by presence of hypoglycemia, clinical signs associated with low glucose concentrations, and resolution of signs after glucose administration or food intake.^44^ Although this traditional approach to diagnosing insulinoma remains useful, advanced imaging techniques and serum insulin measurements are now commonly used to support the diagnosis.^22^ Medical imaging was compatible with insulinoma in 2 thirds of the dogs and histopathological confirmation was obtained in one third of the cases. Insulinomas are rare functional pancreatic β cell neoplasms most commonly reported in medium to large breed dogs,^43^ that secrete excessive insulin in the blood, resulting in hyperinsulinemia-induced hypoglycemia.^26^ Insulin concentrations after feed withdrawal were measured in half of the dogs with presumptive or definitive diagnosis of insulinoma and were increased in the vast majority (80%). Concurrent hypoglycemia (<3.3 mmol/L) and elevated plasma insulin (>60 pmol/L) are characteristic of insulinoma in dogs,^45^ however, plasma insulin levels might remain within the reference range in some cases,^21^^,^^46^ as reported in one dog (20%) in our study. The mean age of dogs with suspected, presumptive or definitive diagnosis of insulinoma in this study was 9.4 years old at clinical onset, consistent with published reports indicating a mean age at diagnosis of around 9 years.^18^^,^^22^^,^^26^

One dog, the youngest individual in the study group (24 months old), was diagnosed with iatrogenic hypoglycemia after administration of fast-acting human insulin in conjunction with a constant rate infusion of 5% glucose for management of concurrent hyperkalemia associated with suspected ischemia–reperfusion injury. Iatrogenic insulin overdose is typically readily identified from the clinical history and might result from either an absolute overdose, such as inadvertent administration of an excessive insulin dose, or a relative overdose, in which a routine insulin dose is administered in the setting of increased glucose utilization or reduced intake (eg, increased physical activity, concurrent illness, or inadequate food intake).^23^ In this present case, an absolute insulin overdose was considered most likely.

The additional presence of generalized tonic–clonic epileptic seizures classifies hypoglycemia-associated myoclonus as more likely epileptic myoclonus.^2^ In half of the cases, myoclonic episodes would progress to generalized tonic–clonic epileptic seizures, supporting an epileptic classification, although the epileptic seizures were most likely reactive in nature. A reactive seizure is a provoked seizure occurring as a natural response from a healthy brain to a transient functional disturbance, such as a metabolic condition (eg, hypoglycemia), which is reversible after correction of the underlying disturbance.^47^ Interpretation is more challenging in the other half of the dogs, in which generalized tonic–clonic epileptic seizures were not observed. While the absence of associated epileptic seizures might suggest nonepileptic myoclonus classification, this does not exclude a cortical origin, and generalized tonic–clonic epileptic seizures might simply not have developed during the period of observation in these cases. Electroencephalogram evaluation during myoclonic episodes would be valuable for distinguishing epileptic from nonepileptic myoclonus and enabling more accurate classification.^2^ Before the hypoglycemia was diagnosed, ASM were administered in 40% of the dogs. However, the myoclonic episodes and epileptic seizures did not improve in any of the cases. In contrast, correction of hypoglycemia resulted in improvement of clinical signs in all dogs, even in the absence of ASM, which were discontinued in all dogs after the diagnosis of hypoglycemia. Although antiseizure therapy is recommended for management of cortical–subcortical myoclonus in humans,^48^ myoclonus associated with metabolic disturbances, such as hypoglycemia, might persist despite ASM administration.^36^^,^^39^ Nevertheless, the retrospective nature of this study prevents more concrete conclusions regarding the efficacy of ASM in these dogs. Information on the duration of ASM treatment before discontinuation was not available, and it is therefore possible that some dogs might have benefited from a longer antiseizure treatment period. Furthermore, some dogs experienced recurrence of signs of neurological disease, including myoclonic episodes and epileptic seizures, despite treatment of the underlying cause of hypoglycemia. Whether adjunctive ASM therapy could have improved long-term control of these signs in relapsing cases remains unknown.

In humans, brain MRI findings in hypoglycemic encephalopathy typically include diffuse cortical diffusion restriction, often accompanied by sulcal effacement and mild diffuse cerebral edema, changes consistent with global metabolic brain injury, commonly associated with hypoglycemia.^39^^,^^49^ Two dogs underwent brain MRI while still exhibiting myoclonic episodes and other signs of neurological disease, including decreased mental status, however, no MRI abnormalities were identified. Both examinations were performed using low-field MRI scans, and diffusion-weighted imaging was not acquired, which might have limited detection of subtle or diffuse metabolic brain lesions in these 2 cases.

All dogs with presumptive of definitive diagnosis of insulinoma that were treated, medically or surgically, improved in the short term, either myoclonic episode free or decreased frequency. Four of them were eventually euthanized later due to clinical deterioration related to the insulinoma. Surgical intervention is generally considered the most effective treatment for canine insulinoma and is associated with prolonged survival,^18^^,^^24^^,^^25^^,^^28^ although the combination with medical treatment seems to prolong survival even more, especially in relapsing cases.^20^ Despite treatment, the overall prognosis remains guarded, as clinical hypoglycemia frequently recurs due to tumor regrowth^21^ and the presence of metastasis.^18^^,^^24^^,^^25^

The main limitations of this study are its retrospective design, its limited number of cases and the inclusion of dogs from multiple facilities, which resulted in variability of diagnostic work-up and treatment protocols. Furthermore, the retrospective nature of this study prevented accurate and consistent assessment of some valuable information, including frequency and duration of myoclonic episodes in most cases. In addition, although myoclonus was observed during the neurological examination in some cases, its diagnosis relied heavily on clinical observations by referring veterinarians and owners, as well as on the evaluation of video recordings. While the progression to generalized tonic–clonic epileptic seizures in some cases would classify these myoclonic episodes as epileptic myoclonus according to the current veterinary classification,^2^ the lack of EEG prevents a more precise classification of the myoclonus in all cases.

In conclusion, myoclonus might represent an underrecognized neurological manifestation of hypoglycemia in dogs, expanding the spectrum of clinical signs, particularly in cases secondary to insulinoma. Measurement of blood glucose concentrations during myoclonic episodes is a fundamental diagnostic approach for identifying hypoglycemia-related myoclonus, especially in older dogs. Medical and surgical treatment might lead to clinical improvement, but duration of response is variable and dependent on the underlying cause. These findings should prompt clinicians to consider hypoglycemia as a differential diagnosis for acute myoclonus in dogs.

## Supplementary Material

Video_1_aalag252

## Off-label antimicrobial declaration

Authors declare no off-label use of antimicrobials.

## Institutional animal care and use committee or other approval declaration

Approval was not required for this study because the animal data analyzed were derived solely from a retrospective review of existing medical records. No additional interventions, treatments, or prospective research procedures were performed beyond routine clinical care. Medical record numbers were recorded prospectively for data collection purposes only, and no activities were undertaken that would have necessitated ethical oversight.

## Abbreviations

ASM: antiseizure medication
CSF: cerebrospinal fluid
CT: computed tomography
MRI: magnetic resonance imaging
